# Network pharmacology and experimental validation to explore the role and potential mechanism of Liuwei Dihuang Decoction in prostate cancer

**DOI:** 10.1186/s12906-024-04572-5

**Published:** 2024-07-26

**Authors:** Xiangyang Zhan, Haoze Li, Jingyun Jin, Xiran Ju, Jiawei Gao, Xinglin Chen, Fuwen Yuan, Jianyi Gu, DongLiang Xu, Guanqun Ju

**Affiliations:** 1 Urology Center, Shuguang Hospital Affiliated to Shanghai University of Traditional Chinese Medicine, Shanghai, 201203 China; 2 Surgical Institute of Integrative Medicine, Shuguang Hospital Affiliated to Shanghai University of Traditional Chinese Medicine, Shanghai, 201203 China; 3 Surgical Institute, Shuguang Hospital Affiliated to Shanghai University of Traditional Chinese Medicine, Shanghai, 201203 China; 4 Shanghai University of Traditional Chinese Medicine, Shanghai, 201203 China

**Keywords:** Liuwei Dihuang Decoction, Prostate cancer, Network pharmacology, Bioinformation analysis, Experimental validation

## Abstract

**Objective:**

To evaluate the anti-tumor effector of Liuwei Dihuang Decoction (LWDHD) in prostate cancer (PCa) and explore the potential mechanism using experimental validation, network pharmacology, bioinformatics analysis, and molecular docking.

**Methods:**

CCK test, Clone formation assay and wound-healing assays were used to determine the effect of LWDHD on prostate cancer growth and metastasis. The active ingredients and targets of LWDHD were obtained from the TCMSP database, and the relevant targets were selected by GeneCards, OMIM and DisGeNET databases for PCa. The cross-targets of drugs and disease were imported into the STRING database to construct protein interactions. The network was also visualized using Cytoscape software and core targets are screened using the Network Analyzer plug-in. The Gene Ontology (GO) and Kyoto Encyclopedia of Genes and Genomes (KEGG) pathway enrichment were analyzed using R software. TCGA database was used to analyze the correlation of bioinformatics genes. AutoDock vina was used to predict the molecular docking and binding ability of active ingredients to key targets. Through WB and q-PCR experiments, the above gene targets were detected to verify the effect of LWDHD on PCa.

**Results:**

CCK and scratch tests confirmed that LWDHD could inhibit the proliferation, invasion and migration of prostate cancer cells. Clone formation experiments showed that LWDHD inhibited the long-term proliferative capacity of PC3 cells. LWDHD and PCa had a total of 99 common targets, establishing a “drug-ingredient-common target” network. Through GO and KEGG enrichment analysis, PI3K/AKT, MAPK, TP53 pathway, MYC, TNF pathway and other signaling pathways were found. Bioinformatics analysis showed that MYC gene was highly expressed and CCND1 and MAPK1 were low expressed in prostate cancer tissues. In addition, TP53, AKT1, MYC, TNF and CCND1 were positively correlated with MAPK1, among which AKT1 and CCND1 were most closely correlated with MAPK1. Molecular docking results showed that quercetin, kaempferol, β-sitosterol and other main active ingredients of LWDHD treatment for PCa were combined with core proteins MAPK1 and AKT1 well. WB and q-PCR results showed that LWDHD inhibited the expression of PI3K and AKT in PC3 cells.

**Conclusion:**

The mechanism of LWDHD therapy for PCa is a multi-target and multi-pathway complex process, which may be related to the biological processes mediated by MAPK1 and AKT1 pathways, such as cell proliferation and inhibition of metastasis, and the regulation of signaling pathways. The PI3K/AKT signaling pathway may be a central pathway of LWDHD to inhibit prostate cancer proliferation.

**Supplementary Information:**

The online version contains supplementary material available at 10.1186/s12906-024-04572-5.

## Background

Prostate cancer (PCa) is one of the most common malignant tumors in men and the fifth leading cause of cancer death in men worldwide [1]. PCa alone accounts for about 13.5% of newly diagnosed male cancer cases and 6.7% of cancer-related death cases globally [2]. The 5-year survival rate of early-stage prostate cancer is as high as 99%, while most prostate cancer is difficult to detect in the early stage [3]. In the stage of metastatic prostate cancer, this proportion is reduced to 28% [4]. This dramatic decline in survival has prompted an urgent need to discover new strategies or targets to prevent prostate cancer acquire progress [5]. Although endocrine therapy and chemoradiotherapy are the basic treatment of PCa, there are still a series of problems such as drug resistance and many adverse reactions [6, 7], which seriously affect the treatment effect of PCa.

Traditional Chinese medicine (TCM) is widely used clinically for the treatment of different types of cancers [8–10]. Liuwei Dihuang Decoction (LWDHD) is a classic recipe, many studies have shown that it has an inhibitory effect on the occurrence and development of cancer [11]. For example, LWDHD can inhibit triple-negative breast cancer metastasis by regulating Wnt pathway and interfering with catenin/T cytokine interaction [12]. LWDHD can inhibit the occurrence of gastric cancer in diabetic mice [13]. Clinical practices have found that compared with endocrine therapy alone, combined treatment with LWDHD can prolong the overall survival time (OS) and the time to progression of PSA progression (TTPP) in patients with advanced prostate cancer [14, 15]. However, Experimental studies on the treatment of prostate cancer by LWDHD are still lacking. Otherwise, due to the complex composition and target of TCM relapse [16], it is difficult to elucidate the molecular mechanism of LWDHD for the treatment of PCa.

Network pharmacology (NP), based on the “disease-gene-target-drug” interaction network, reveals the intervention and influence of drugs on the disease network through the analysis of genes, proteins, diseases, drugs and other information in the existing database, thus displaying the synergistic effect of drugs on the human body [17, 18]. It is suitable for studying the relationship between various TCM drug components and disease targets [19]. Therefore, through network pharmacology and biological information analysis combined with molecular docking technology, we explained the mechanism of action of LWDHD in the treatment of PCa, Otherwise, After lyze-dried powder of LWDHD was used to treat PCa, the proliferation ability of PCa cells was detected by CCK8 method, and the effect of LWDHD on the motor ability of PCa cells was detected by cell scratch test, and the related pathways were verified by q-PCR and WB experiments. In order to provide reference for further exploration of its clinical, experimental, and data mining.

## Materials and methods

### Cells

Prostate cancer PC3 cell line was purchased from the Dalian Meilun Biotech Co., Ltd.

### Preparation of LWDHD

LWDHD contains 6 TCM formulations: *Rehmanniae Radix Praeparata*(Shudihuang, 15 g), *Rhizoma Dioscoreae*(Shangyao, 10 g), *Cornus Officinalis Sieb. Et Zucc.*(Shanzhuyu, 10 g), *Poria Cocos(Schw.) Wolf.*(Fuling, 10 g), *Cortex Moutan*(Mudanpi, 9 g) and *Alisma Orientale (Sam.) Juz.*(Zexie, 10 g). All herbs were purchased by Shanghai Hongqiao Chinese Herbal Medicine Co., Ltd. and authenticated by Professor FUwen Yuan of Shanghai University of Traditional Chinese Medicine. The preparation of dosage forms is controlled by the quality of Shuguang Hospital Affiliated to Shanghai University of Traditional Chinese Medicine. Each decoction was soaked in water for 30 min and decocted for 45 min. Then, filtered and collected the medicinal juice, and repeated twice. Finally concentrated all filtrate and freeze-dried for the following experiments.

### Cell viability assays

Cell viability was assessed by the CCK-8 assay kit (B34304, Bimake, USA) as per the manufacturer’s instructions. Briefly, cells were seeded onto 96-well plates at a density of 6000 cells/well in 96-well plates for 24 h. Next, the indicated doses of LWDHD were added and cultured for another 72 h. Cells were then cultured in 90 µL fresh medium and 10 µL CCK-8 reagent for another 1 h. The absorbance at 450 nm was read using a 96-well plate reader (Fluostar Omega Spectrofluorometer, BMG Technologies, Offenburg, Germany).

### Wound-healing assays

The cell migration was determined with wound-healing assays. Cells (1 × 10^6^ cells/well for PC3) were plated in six-well plates. After 24 h, the wound was created using 10 µl tips. The confluent cells were changed into FBS-free medium containing 20 µg/mL of indicated drugs, which has no significant effect on cell growth. The cell migration status toward the wound was photographed (×10) (Olympus America Inc.) after 0 h, 24 h, and 48 h, respectively.

### Colony formation

The colony formation assay was performed to examine the effect of LWDHD on cell colony survival. PC3 cell were seeded into 6-well plates and cultured overnight. Different concentrations of LWDHD were added to each well. After that, the cell culture medium was changed and maintained with the same dose of compounds every 3 days until the colonies were visible. The cells were fixed using 4% paraformaldehyde and stained with crystal violet staining solution in around 14 days.

### Active ingredients and targets screening of LWDHD

By TCMSP database (https://tcmspw.com/tcmsp.php) to retrieve shudihuang, shanyao, shanzhuyu, fuling, mudanpi, and zexie, the active ingredient, filter condition for “OB ≥30%, DL ≥0.18”. The targets of active ingredients were predicted by TCMSP database. symbol correction of Uniprot database (https://www.uniprot.org/) was performed on all targets to remove any that do not correspond.

### Screening of target genes for prostate cancer

With “Prostate cancer” as keywords, using GeneCards (http://www.genecards.org/), OMIM database (https://www.omim.org/) and DisGeNET database (https://www.disgenet.org/) for disease genes. Among them, GeneCards database’s target screening condition was “Relevance score > 20”, in order to obtain highly relevant targets. Using jvenn (http://jvenn.toulouse.inra.fr/app/example.html) online tool to map mutual targets for LWDHD and prostate cancer.

### Construction of “drug-ingredient-target” network diagram

The network diagram of “drug-ingredient-target” was drawn with Cytoscape3.9.0 software, and topological analysis was carried out with network Analyzer plug-in to obtain core compounds according to degree value.

### Construction of protein interaction network

The common targets of drugs and disease were input into the STRING database (https://string-db.org/cgi/input.pl) to obtain the protein interaction network (PPI), in which the biological species was “Homo sapiens”, and the protein interaction score was ≥ 0.70. PPI Network was imported into Cytoscape3.9.0 software, Network Analyzer plug-in was used for topological analysis, and top 10 genes were selected as key targets according to degree. In addition, the common targets of drugs and disease were imported into metascape database, and cluster module analysis of MCODE was carried out for genes, so as to intuitively show the interaction between targets.

### GO and KEGG enrichment analysis

Enrichment analysis and visualization of biologicalprocess (BP), molecular function (MF), cellular component (CC) of GO and KEGG pathways were carried out for common targets of drugs and diseases with the help of R packages such as “clusterProfiler”, and “p value < 0.05,q value < 0.05” were set. The pathways associated with prostate cancer were screened using KEGG database (https://www.kegg.jp/), and the "pathway-target" network diagram was drawn. The core targets associated with the pathway were obtained after topological analysis of the network diagram.

### Gene analysis

RNAseq data (level3) and corresponding clinical information for prostate cancer were obtained from TCGA database (https://portal.gdc.com). Correlation analysis methods of expression distribution of related genes were implemented by R v4.0.3, and multi-gene correlation maps were displayed by R software.

### Molecular docking

Determine the compound name, molecular weight and 2D structure of the active ingredients from the PubChem database, then download the 3D structure of the proteins from the RCSBPDB database (http://www.rcsb.org/). Then, AutoDock software (http://vina.scripps.edu/) was used to prepare ligands and proteins required for molecular docking. For target proteins, the crystal structure need to be pretreated, including removing water molecules, hydrogenating, modifying amino acids, optimizing energy and adjusting force field parameters. After that, vina inside pyrx software was used for virtual docking screening, and its Binding Affinity (kcal/mol) value represents the binding ability of the two. The lower the binding ability, the more stable the binding between ligand and receptor is. Finally, Discovery Studio is used for analysis. PYMOL software was used for drawing. Protein accession number: CCND1 (6P8F)、MAPK1 (3I5Z)、MYC (6G6K)、TNF (5UUI)、TP53 (IKZY)、AKT1 (6HHG).

### RT-qPCR

For all RT-qPCR, 250,000 cells were collected per sample. RNA extraction was performed using the RNA Clean and Concentrator-5 kit (Zymo R1017). Total RNA was reverse transcribed to cDNA using the SuperScript VILO cDNA Synthesis Kit (ThermoFisher 11,756,050). cDNA was used for qPCR using the Fast SYBR Green Master Mix (ThermoFisher 4,385,612) and uniquely designed gene expression probes (IDT), including a GAPDH reference gene, in three technical replicates per reaction.

### Protein extraction and western blotting

Whole-cell lysates were obtained from cells as previously described (24). Briefly, cells were washed with PBS, collected with radioimmunoprecipitation assay (RIPA) lysis buffer containing 1% Nonidet P-40, 0.5% sodium deoxycholate, and 0.1% sodium dodecyl sulfate. For chromatin fraction, cells were washed twice with PBS and treated with CSK buffer for 5 min on ice followed by washing with PBS three times. Cell lysates were collected with RIPA lysis buffer. WB samples were resolved by SDS-PAGE and analyzed by standard WB protocols. Signals of the western blots were detected by a standard chemiluminescence reagent (Cytivia) and analyzed using a ChemiDoc system (Bio-Rad Laboratories) using indicated antibodies.

### Statistical methods

Statistical analysis was conducted using SPSS 28 software. Continuous variables were denoted as “XS”. One-way analysis of variance (ANOVA) was employed for comparing multiple groups, and pairwise comparisons were performed using the least significant difference (LSD) t-test. A significance level of *P* < 0.05 was considered statistically significant, while a significance level of *P* < 0.01 was considered highly statistically significant.

### The effects of LWDHD on prostate cancer cells

**Fig. 1:**
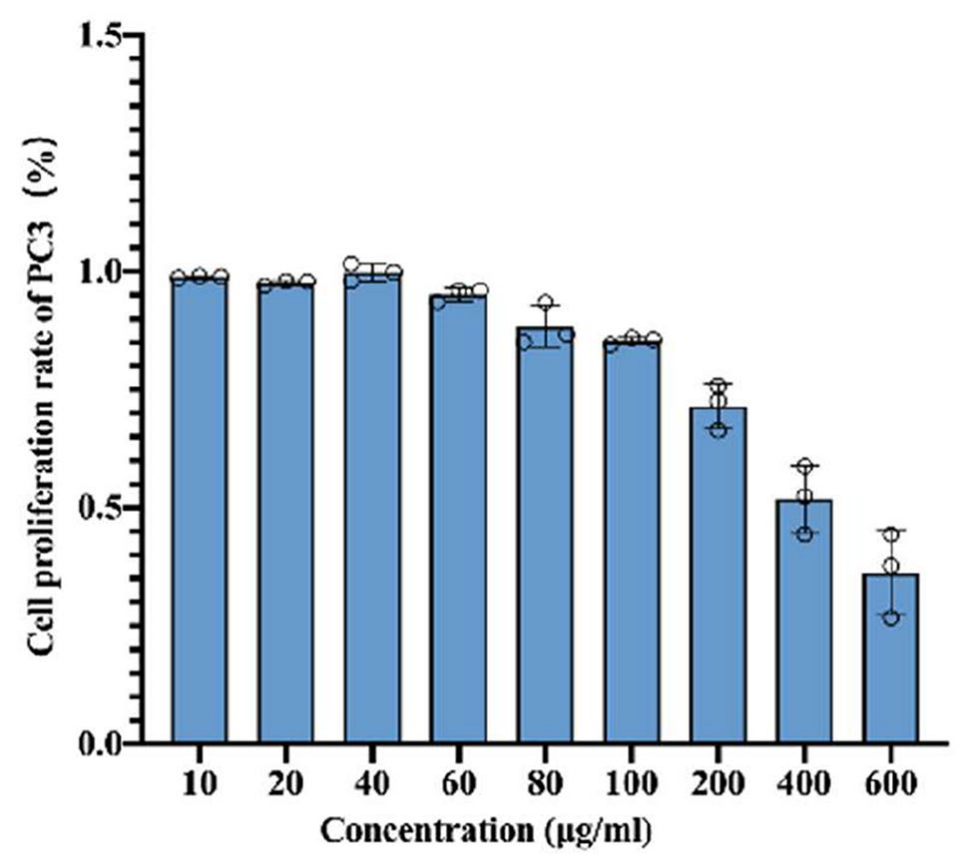
Cell proliferation experiment analyzing the effects of LWDHD at different concentrations on the proliferation capacity of prostate cancer cells. The effects of LWDHD on the proliferation of PC3 cells (*n* = 3, XS)

In order to investigate the effects of LWDHD on prostate cancer, we intervened PC3 cells with different concentrations of LWDHD (0 µg/mL, 10 µg/mL, 20 µg/mL, 40 µg/mL, 60 µg/mL, 80 µg/mL, 100 µg/mL, 200 µg/mL, 400 µg/mL, and 600 µg/mL) for 48 h. The cell proliferation inhibition rate was measured using the CCK-8 assay. The results showed that the concentration of LWDHD was negatively correlated with the proliferation rate of PC3 cells and positively correlated with the inhibition rate (Fig. 1).

**Fig. 2:**
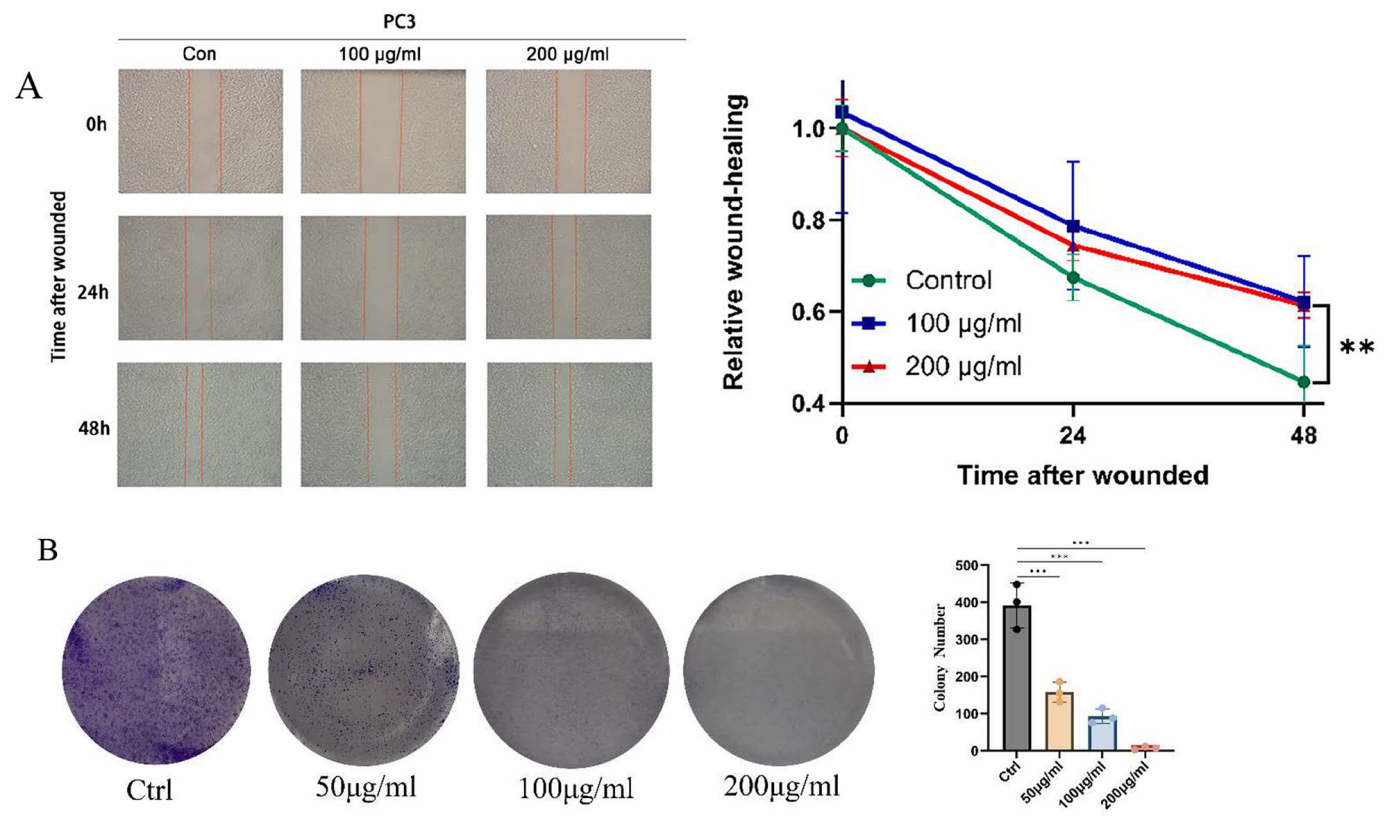
(**A**) The scratch assay is performed to analyze the effects of different concentrations of LWDHD on the migratory capability of PC3 cells. (**B**) Effects of different concentrations of LWDHD on clone formation in PC3 cells

Furthermore, based on the aforementioned cell experiment results, we conducted a scratch assay to assess the effects of LWDHD at different concentrations (100 µg/mL, 200 µg/mL) on the invasive and migratory capabilities of PC3 cells after 24 h and 48 h of intervention. The results revealed that compared to the CON group, the LWDHD group significantly reduced the healing ability of PC3 cells and inhibited the migration capacity of prostate cancer cells, with a highly significant statistical difference (*P* < 0.01) (Fig. 2A). Therefore, this indicates that LWDHD can suppress the proliferation of prostate cancer cells and impede their invasive and metastatic behavior. Clone formation experiments demonstrated that LWDHD dose-dependently inhibits the long-term proliferative capacity of prostate cancer (Fig. 2B).

### LWDHD effective active ingredients

Through TCMSP database screening of active ingredients, the screening conditions were “OB ≥ 30%, DL ≥ 0.18”, 74 active ingredients were obtained. There were 558 corresponding targets of active ingredients (Table 1). The uniprot database was used to standardize the drug targets and 190 drug targets were obtained.

**Table 1:** The main active ingredients of LWDHD

Chinese name	Latin name	MOL ID	Molecule name	OB(%)	DL
Shudihuang	Rehmannia glutinosa	MOL000449	Stigmasterol	43.83	0.76
		MOL000359	Sitosterol	36.91	0.75
Shanyao	Dioscoreae Rhizoma	MOL001559	Piperlonguminine	80.88	0.18
		MOL001736	(-)-taxifolin	64.01	0.27
		MOL000310	Denudatin B	61.47	0.38
		MOL000322	Kadsurenone	60.51	0.38
		MOL005429	Hancinol	59.05	0.37
		MOL005430	Hancinone C	54.72	0.39
		MOL005435	24-Methylcholest-5-enyl-3belta-O-glucopyranoside_qt	45.33	0.72
		MOL005438	Campesterol	43.83	0.71
		MOL005440	Isofucosterol	43.78	0.76
		MOL000449	Stigmasterol	38.16	0.76
		MOL005458	Dioscoreside C_qt	37.87	0.87
		MOL000546	Diosgenin	37.58	0.81
		MOL005461	Doradexanthin	37.58	0.54
		MOL005463	Methylcimicifugoside_qt	36.38	0.24
		MOL005465	AIDS180907	31.69	0.77
		MOL000953	CLR	30.71	0.68
Shanzhuyu	Cornus officinalis Sieb. et Zucc	MOL005531	Telocinobufagin	69.99	0.79
		MOL005552	Gemin D	68.83	0.56
		MOL005360	Malkangunin	57.71	0.63
		MOL005486	3,4-Dehydrolycopen-16-al	46.64	0.49
		MOL001495	Ethyl linolenate	46.1	0.2
		MOL005557	Lanosta-8,24-dien-3-ol,3-acetate	44.3	0.82
		MOL000449	Stigmasterol	43.83	0.76
		MOL002879	Diop	43.59	0.39
		MOL001494	Mandenol	42	0.19
		MOL005503	Cornudentanone	39.66	0.33
		MOL001771	Poriferast-5-en-3beta-ol	36.91	0.75
		MOL000358	Beta-sitosterol	36.91	0.75
		MOL000359	Sitosterol	36.91	0.75
		MOL005530	Hydroxygenkwanin	36.47	0.27
		MOL005481	2,6,10,14,18-pentamethylicosa-2,6,10,14,18-pentaene	33.4	0.24
		MOL008457	Tetrahydroalstonine	32.42	0.81
		MOL002883	Ethyl oleate (NF)	32.4	0.19
		MOL003137	Leucanthoside	32.12	0.78
		MOL005489	3,6-Digalloylglucose	31.42	0.66
		MOL000554	Gallic acid-3-O-(6’-O-galloyl)-glucoside	30.25	0.67
Mudanpi	Cortex Moutan	MOL001925	Paeoniflorin_qt	68.18	0.4
		MOL007369	4-O-methylpaeoniflorin_qt	67.24	0.43
		MOL007384	Paeonidanin_qt	65.31	0.35
		MOL000211	Mairin	55.38	0.78
		MOL000492	(+)-catechin	54.83	0.24
		MOL000098	Quercetin	46.43	0.28
		MOL007374	5-[[5-(4-methoxyphenyl)-2-furyl]methylene]barbituric acid	43.44	0.3
		MOL007382	Mudanpioside-h_qt 2	42.36	0.37
		MOL000422	Kaempferol	41.88	0.24
		MOL000359	Sitosterol	36.91	0.75
		MOL007003	Benzoyl paeoniflorin	31.14	0.54
Fuling	Poria	MOL000300	Dehydroeburicoic acid	44.17	0.83
		MOL000282	Ergosta-7,22E-dien-3beta-ol	43.51	0.72
		MOL000283	Ergosterol peroxide	40.36	0.81
		MOL000275	Trametenolic acid	38.71	0.8
		MOL000287	3beta-Hydroxy-24-methylene-8-lanostene-21-oic acid	38.7	0.81
		MOL000285	(2R)-2-[(5R,10 S,13R,14R,16R,17R)-16-hydroxy-3-keto-4,4,10,13,14-pentamethyl-1,2,5,6,12,15,16,17-octahydrocyclopenta[a]phenanthren-17-yl]-5-isopropyl-hex-5-enoic acid	38.26	0.82
		MOL000292	Poricoic acid C	38.15	0.75
		MOL000279	Cerevisterol	37.96	0.77
		MOL000296	Hederagenin	36.91	0.75
		MOL000276	7,9(11)-dehydropachymic acid	35.11	0.81
		MOL000289	Pachymic acid	33.63	0.81
		MOL000280	(2R)-2-[(3 S,5R,10 S,13R,14R,16R,17R)-3,16-dihydroxy-4,4,10,13,14-pentamethyl-2,3,5,6,12,15,16,17-octahydro-1 H-cyclopenta[a]phenanthren-17-yl]-5-isopropyl-hex-5-enoic acid	31.07	0.82
		MOL000273	(2R)-2-[(3 S,5R,10 S,13R,14R,16R,17R)-3,16-dihydroxy-4,4,10,13,14-pentamethyl-2,3,5,6,12,15,16,17-octahydro-1 H-cyclopenta[a]phenanthren-17-yl]-6-methylhept-5-enoic acid	30.93	0.81
		MOL000290	Poricoic acid A	30.61	0.76
		MOL000291	Poricoic acid B	30.52	0.75
Zexie	Alisma plantago-aquatica	MOL002464	1-Monolinolein	37.18	0.3
		MOL000359	Sitosterol	36.91	0.75
		MOL000853	Alisol B	36.76	0.82
		MOL000831	Alisol B monoacetate	35.58	0.81
		MOL000862	[(1 S,3R)-1-[(2R)-3,3-dimethyloxiran-2-yl]-3-[(5R,8 S,9 S,10 S,11 S,14R)-11-hydroxy-4,4,8,10,14-pentamethyl-3-oxo-1,2,5,6,7,9,11,12,15,16-decahydrocyclopenta[a]phenanthren-17-yl]butyl] acetate	35.58	0.81
		MOL000830	Alisol B	34.47	0.82
		MOL000856	Alisol C monoacetate	33.06	0.83
		MOL000854	Alisol C	32.7	0.82
		MOL000832	Alisol, b,23-acetate	32.52	0.82
		MOL000849	16β-methoxyalisol B monoacetate	32.43	0.77

### Potential targets of LWDHD in correlated with prostate cancer

**Fig. 3:**
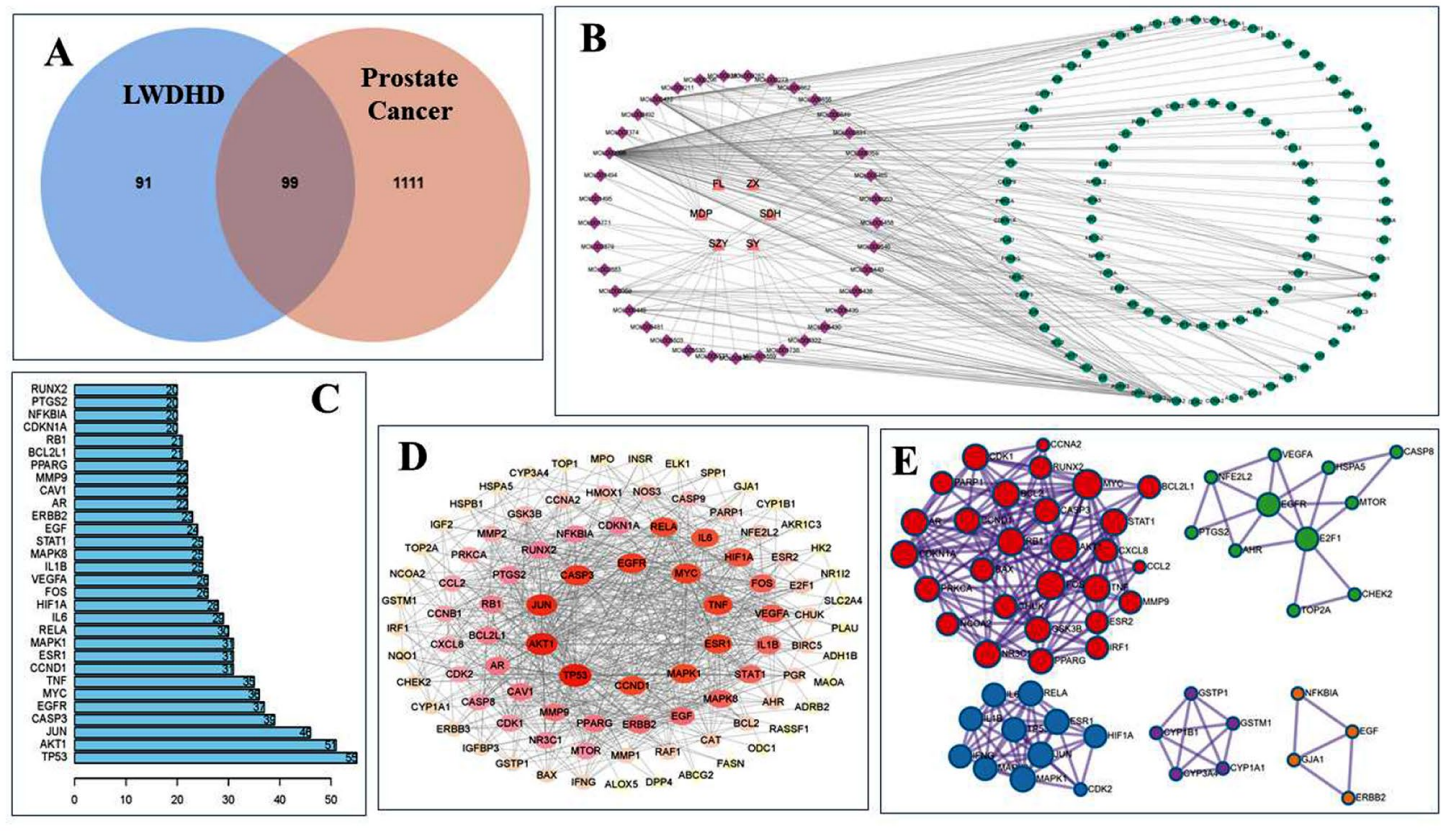
(**A**) The Venn diagram of potential targets for the treatment of prostate cancer by LWDHD. (**B**) The network diagram of “drug-ingredient-target”. The orange triangle is composed of LWDHD, the purple diamond shape is the active ingredient of LWDHD, and the green circle is the target of LWDHD for the treatment of prostate cancer. (**C**) Histogram of degree values for potential targets of LWDHD for the treatment of prostate cancer. (**D**) PPI Network Topology Analysis (The redder the color, the larger the shape, the higher the degree value). (**E**) The cluster analysis based on MCODE. Red: MCODE1; Blue: MCODE2; Green: MCODE3; Purple: MCODE4; Orange: MCODE5

Targets of prostate cancer were obtained from GeneCards database, OMIM database and Disgenet database. There were 12,377 related targets in GeneCards database, and 692 of them had Relevance score > 20. 168 targets were obtained from OMIM database. The DisGENET database yielded 683 targets. A total of 1210 prostate cancer-related targets were obtained by deweighting the three databases. 190 LWDHD drug targets and 1210 prostate cancer targets were imported into jvenn online webpage tool to draw Venn diagram, and 99 common targets were obtained (Fig. 3A).

### Construction of “drug-ingredient-target” network and screening of core ingredients

Using Cytoscape3.9.0 software rendering “drug-ingredient-target” network diagram (Fig. 3B), including 142 nodes and 257 wire, with the aid of network Analyzer plug-in topology analysis was carried out on the network diagram, higher degree of the top three of quercetin, kaempferol, beta-sitosterol, corresponding 83, 31 and 14 targets respectively, suggesting that these three active ingredients were the main material basis of LWDHD in the treatment of prostate cancer.

### Construction of PPI network and screening of key targets

The 99 intersection targets were imported into the STRING database, and the obtained PPI relationship was imported into Cytoscape3.9.0 software, and the bar graph of PPI was visualized with R software (Fig. 3C and D). PPI network contains 94 nodes and 717 edges. Topology analysis was carried out using network Analyzer plug-in. The top 10 genes of the degree value were TP53, AKT1, JUN, CASP3, EGFR, MYC, TNF, CCND1, MAPK1 and ESR1. These results suggested that these targets may be the key targets of LWDHD. In addition, 99 common targets of drugs and disease were imported into metascape database, and MCODE cluster module analysis was performed on the genes (Fig. 3E). A total of 5 cluster modules were enriched, suggesting that LWDHD may inhibit the occurrence and development of prostate cancer through tumor pathway, IL-18 signaling pathway, Th17 cell differentiation and other pathways (Table 2).

**Table 2:** The detailed information table of mcode cluster analysis

Class cluster	Target spot	The function of genes dominated by cluster was TOP3(Sort by log10(*P*))
MCODE1	CCNA2, CDK1, PARP1, AR, CDKN1A, PRKCA, NCOA2, NR3C1, PPARG, IRF1, ESR2, MMP9, CCL2, CXCL8, STAT1, BCL2L1, MYC, RUNX2, BCL2, CCND1, BAX, CHUK, GSK3B, TNF, AKT1, CASP3, RB1, FOS	GO: hsa05200 cancer pathway (-28.8); GO: hsa05161 hepatitis B (-26.8); GO: WP4754 IL-18 Signaling Pathway (-25.3)
MCODE2	IL6, IL1B, IFNG, MAPK2, MAPK1, CDK2, HIF1A, ESR1, RELA, TP53, JUN	GO: hsa04659 Th17 cell differentiation (-17.5); GO: hsa05200 tumor pathway (-16.6); GO: M115 PID REG GR path (-15.6)
MCODE3	VEGFA, NFE2L2, PTGS2, AHR, EGFR, HSPA5, CASP8, MTOR, E2F1, TOP2A, CHEK2	GO: WA2586 Aromatic hydrocarbon receptor network pathway (-14.3); GO: WP1984 Breast Cancer Integrated Therapy Pathway (-13.6); GO: WP4674 Squamous Cell carcinoma of head and neck (-13.0)
MCODE4	GSTP1, CYP1B1, CYP3A4, CYP1A1, GSTM1	GO: hsa05204 Chemical carcinogenicity -DNA adduction (-13.3); GO: Metabolism of exogenous substances by cytochrome P450 of hsa00980 (-13.0); GO: 0006805 Heterologous metabolic processes (-12.3)
MCODE5	NFKBIA, GJA1, EGF, ERBB2	GO: hsa05215 Prostate cancer (-6.9); GO: WP437 EGF/EGFR signal path (-6.2); GO: hsa05200 Tumor Pathway (-4.7)

### GO and KEGG pathway enrichment analysis

**Fig. 4:**
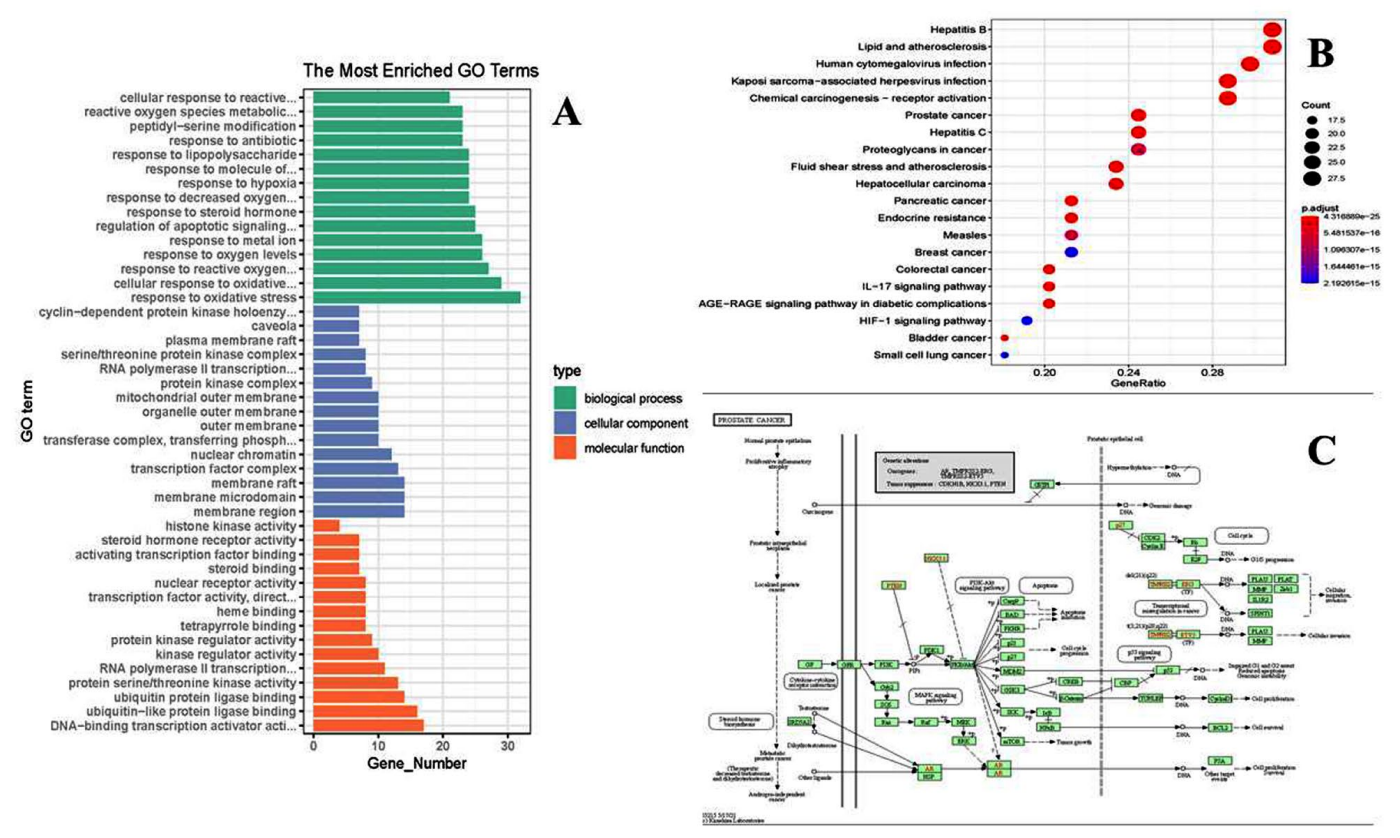
(**A**) The histograms of GO enrichment analysis. (**B**) The bubble map of KEGG enrichment pathway. (**C**) Prostate cancer-related pathways based on the KEGG database

The R software was used to perform GO and KEGG enrichment analysis and visualization of 99 common targets of drugs and disease, and a total of 2142 GO entries were obtained, and 1980 BP entries were obtained, including oxidative stress response, reactive oxygen species response, regulation of apoptosis signaling pathway, etc. There were 35 items of CC, including cytoplasmic membrane, nuclear chromatin, membrane raft, etc. There were 127 items of MF, including RNA polymerase II transcription, heme binding, steroid binding, etc. The first 15 pieces of information for the three items were shown in Fig. 4A. In addition, KEGG enriched 164 pathways, and the top 20 pathways closely related to prostate cancer were shown in Fig. 4B; Table 3. The mechanism of LWDHD in the treatment of prostate cancer mainly acts on *TP53, AKT1, MYC, TNF* signaling pathways, etc.

**Table 3:** KEGG enrichment pathway (based on gene number)

ID	Pathway	Number of enriched genes
hsa05161	Hepatitis B	29
hsa05417	Lipid and atherosclerosis	29
hsa05163	Human cytomegalovirus infection	28
hsa05167	Kaposi sarcoma-associated herpesvirus infection	27
hsa05207	Chemical carcinogenesis - receptor activation	27
hsa04151	PI3K-Akt signaling pathway	26
hsa05165	Human papillomavirus infection	25
hsa05215	Prostate cancer	23
hsa05160	Hepatitis C	23
hsa05205	Proteoglycans in cancer	23
hsa04010	MAPK signaling pathway	23
hsa05418	Fluid shear stress and atherosclerosis	22
hsa05225	Hepatocellular carcinoma	22
hsa05169	Epstein-Barr virus infection	22
hsa05166	Human T-cell leukemia virus 1 infection	22
hsa05208	Chemical carcinogenesis - reactive oxygen species	22
hsa05206	MicroRNAs in cancer	21
hsa05212	Pancreatic cancer	20
hsa01522	Endocrine resistance	20

**Fig. 5:**
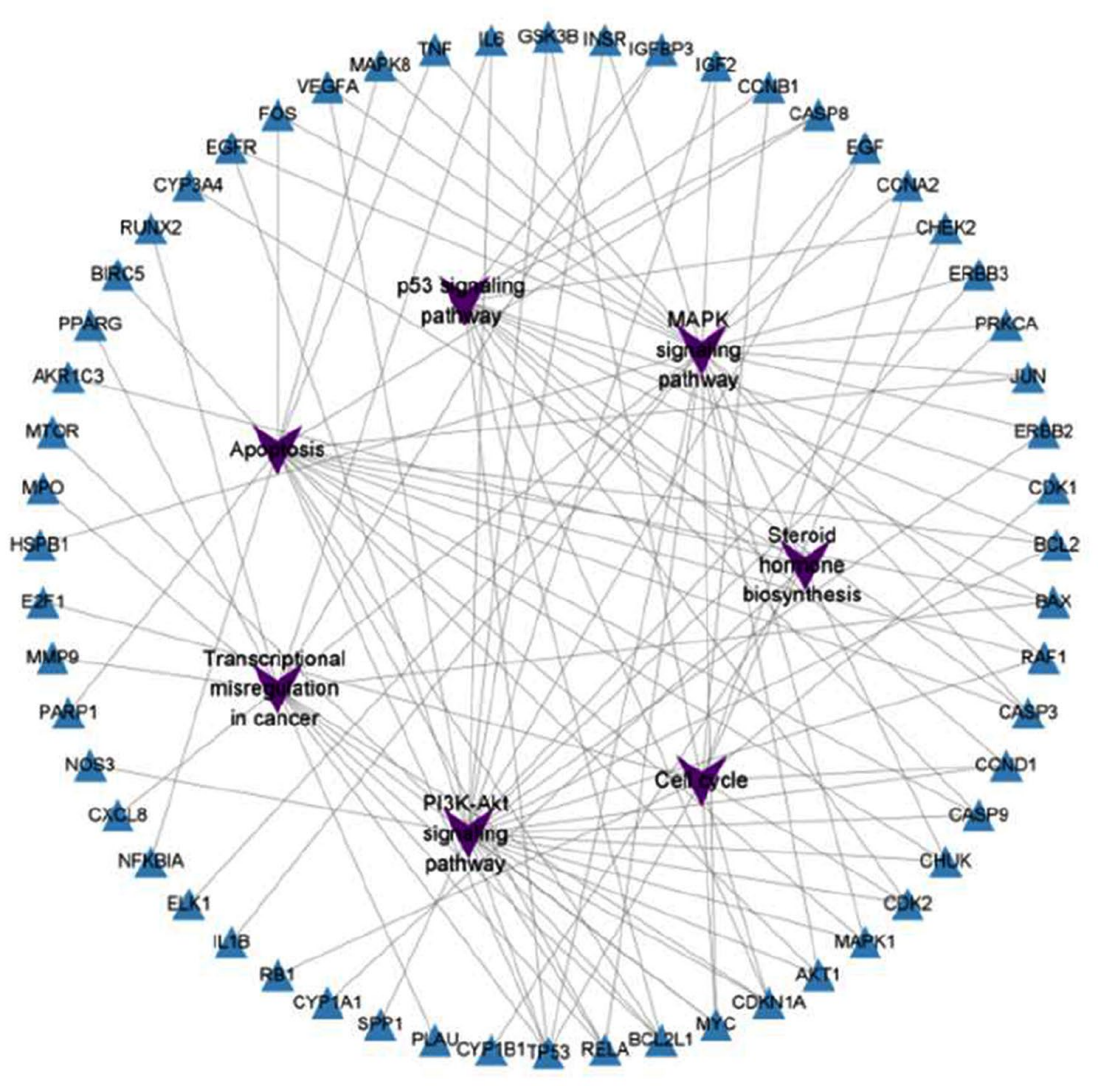
The network diagram of “Pathway-Target”. The purple quadrilateral represents the screened pathway, and the blue triangle represents the enriched targets of the screened pathway

According to KEGG database, there were 8 pathways related to prostate cancer (Fig. 4C). By comparing our results, we can see that there were 7 pathways related to the treatment of prostate cancer by LWDHD, namely, MAPK pathway, PI3K/AKT pathway, P53 pathway, immune escape pathway, tumor transcription regulation pathway, cell circulation pathway, and steroid hormone biosynthesis pathway. The “pathway-target” network diagram was constructed based on the target information enriched in the pathway, and imported into cytoscape software for visualization analysis (Fig. 5). The network contains 63 nodes and 113 edges. According to topological analysis, the top 10 core targets were TP53, MYC, CDKN1A, BCL2L1, RELA, CCND1, CDK2, AKT1, BCL2 and MAPK1. Compared with the top 10 core targets of PPI network, there were 6 common targets, namely TP53, AKT1, MYC, TNF, CCND1, and MAPK1, which may be important targets for the therapeutic effect of LWDHD.

### Gene correlation analysis

**Fig. 6:**
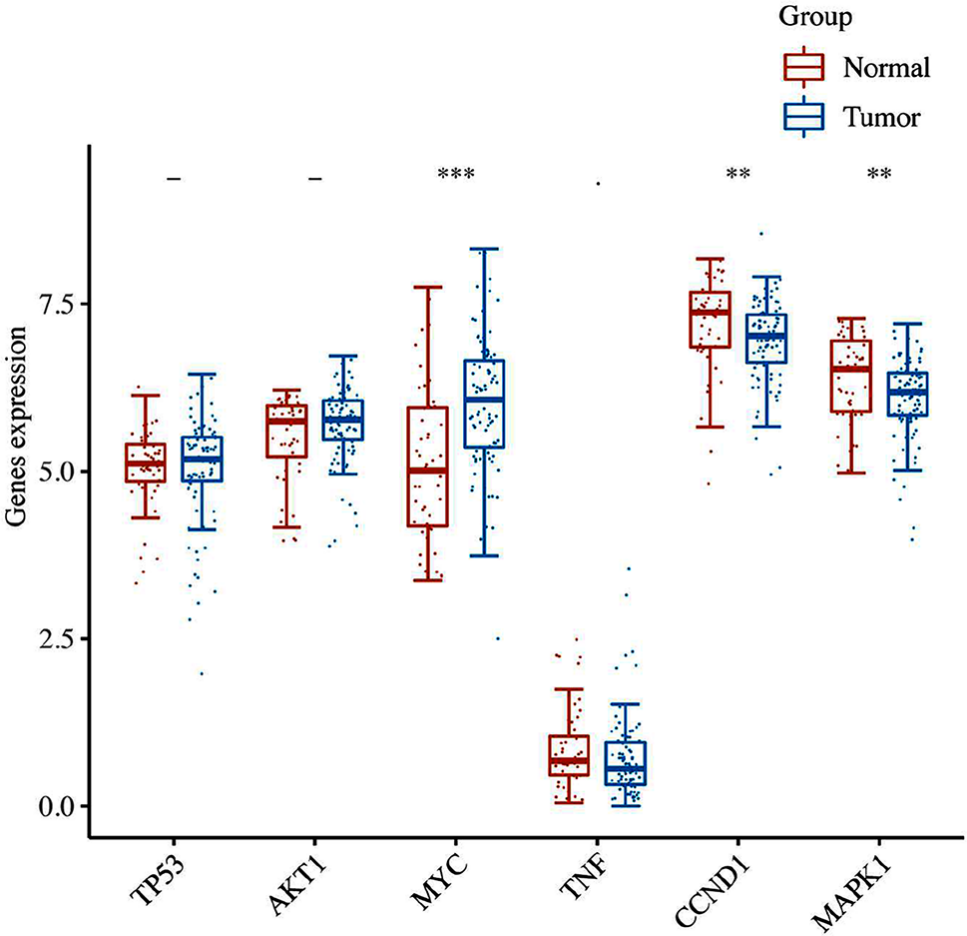
Gene expression in tumor and normal tissues. The expression distribution of genes in 92 tumor tissues and 52 normal tissues, in which the horizontal coordinate represents different genes, and the vertical coordinate represents the expression distribution of related genes, in which different colors represent different groups, *p < 0.05, **p < 0.01, ***p < 0.001, asterisk represents importance (*p). The significance of two groups of samples was determined by wilcox test, and the significance of three groups was determined by Kruskal-Wallis test

**Fig. 7:**
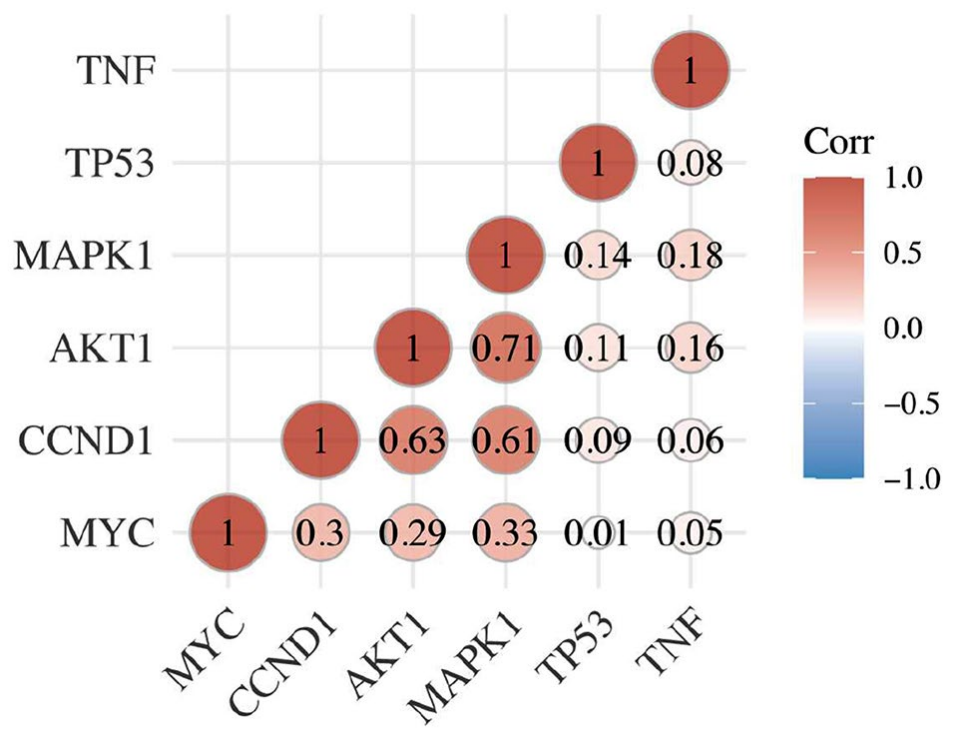
Multiple genes and multiple correlation heat maps. Abscissa and ordinate represents genes, with different color represents the correlation coefficient (red for positive correlation, blue represents negative correlation), the deeper the color represents the stronger the correlation between them

Through TCGA database to obtain the RNAseq data for prostate cancer (level3) and corresponding clinical information. And R software was used to analyze the gene expression of TP53, AKT1, MYC, TNF, CCND1 and MAPK1, the main targets of LWDHD in the treatment of prostate cancer. It was found that MYC gene was highly expressed in prostate cancer tissues, while CCND1 and MAPK1 were lowly expressed in prostate caner tissues compared with adjacent tissues (Fig. 6). In addition, our gene correlation analysis showed that TP53, AKT1, MYC, TNF, CCND1 and MAPK1 all had a certain positive correlation, and the correlation between AKT1, CCND1 and MAPK1 was the closest (Fig. 7). In conclusion, high expression of MYC and low expression of CCND1 and MAPK1 may be related to the occurrence of prostate cancer and the association between TP53, AKT1, MYC, TNF, CCND1 and MAPK1 may accelerate this process.

#### Molecular docking

**Fig. 8:**
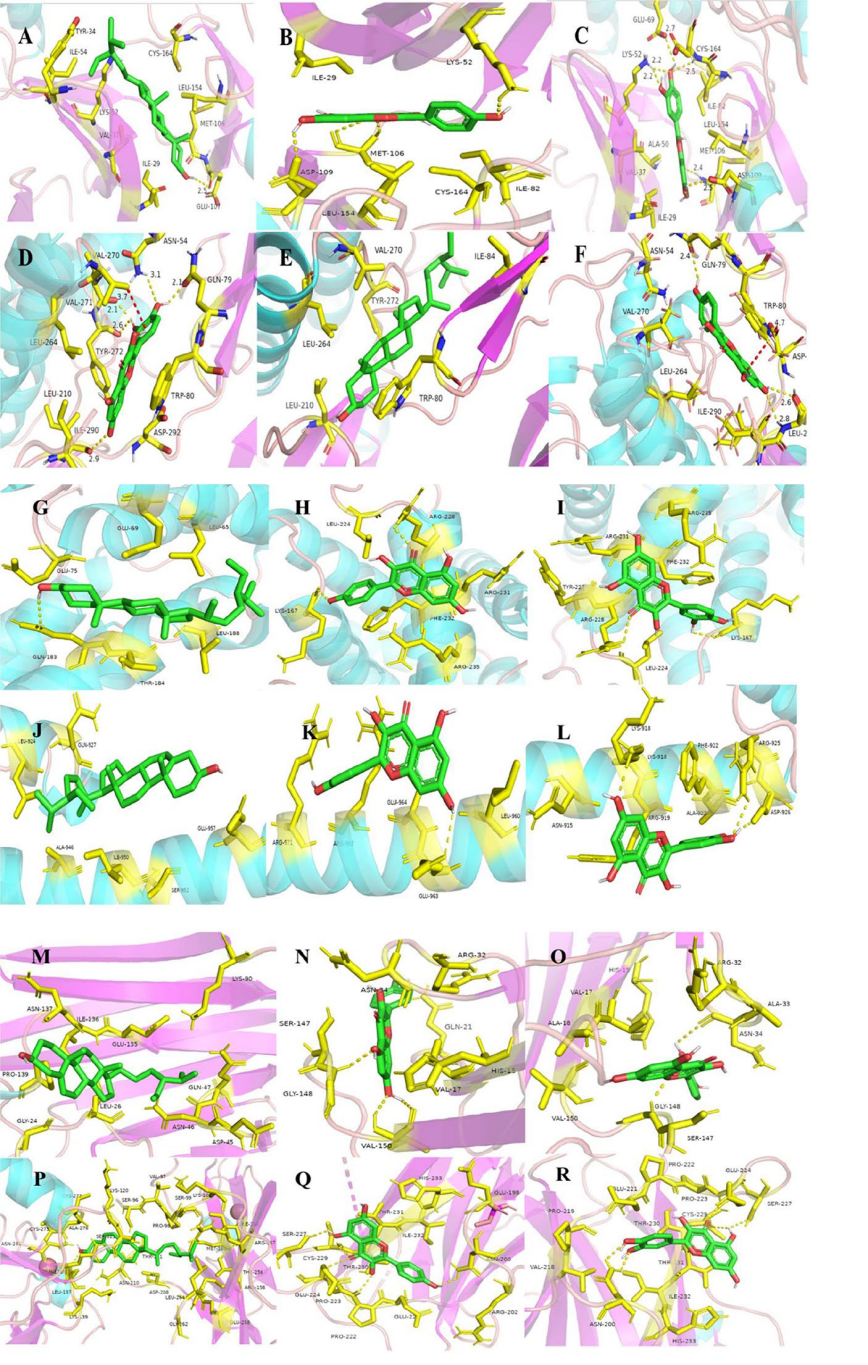
Molecule docking diagram of main components and core target of LWDHD in the treatment of prostate cancer. (**A**) beta-Sitosterol with MAPK1; (**B**) Kaempferol with MAPK1; (**C**) Quercetin with MAPK1; (**D**) Quercetin with AKT1; (**E**) beta-Sitosterol with AKT1; (**F**) Kaempferol with AKT1; (**G**) beta-Sitosterol with CCND1; (**H**) Kaempferol with CCND1; (**I**) Quercetin with CCND1; (**J**) beta-Sitosterol with MYC; (**K**) Kaempferol with MYC; (**L**) Quercetin with MYC; (**M**) beta-Sitosterol with TNF; (**N**) Kaempferol with TNF; (**O**) Quercetin with TNF; (**P**) beta-Sitosterol with TP53; (**Q**) Kaempferol with TP53; (**R**) Quercetin with TP53

AutoDock software was used for molecular docking of quercetin, kaempferol, beta-sitosterol found in the above analysis with six selected targets (TP53, AKT1, MYC, TNF, CCND1 and MAPK1), and Discovery Studio was used for analysis. The lower the binding energy between ligand and receptor, the more likely they were to interact. The binding energies of quercetin with AKT1, MAPK1, TP53, TNF, CCND1 and MYC is -9.4, -9.0, -7.3, -7.0, -6.6 and − 5.9 kcal/mol, respectively. The binding energies of kaempferol with AKT1, MAPK1, TP53, CCND1, TNF and MYC is -9.0, -8.7, -7.1, -6.9, -6.6 and − 5.9 kcal/mol, respectively. The binding energies of beta-sitosterol with AKT1, MAPK1, TP53, CCND1, MYC and TNF is -10.7, -9.7, -7.1, -6.7, -6.5 and − 6.4 kcal/mol, respectively. As shown in Table 4; Fig. 8.

**Table 4:** Molecular docking of compounds and targets in LWDHD treatment of prostate cancer

	CCND1	MAPK1	MYC	TNF	TP53	AKT1
beta-Sitosterol	-6.7	-9.7	-6.5	-6.4	-7.1	-10.7
Kaempferol	-6.9	-8.7	-5.9	-6.6	-7.1	-9
Quercetin	-6.6	-9	-5.9	-7	-7.3	-9.4

*Note*: Drug binding energy using PyRx docking software, in kcal/mol

#### LWDHD can suppress prostate cancer proliferation by inhibiting the PI3K/AKT signaling pathway

**Fig. 9:**
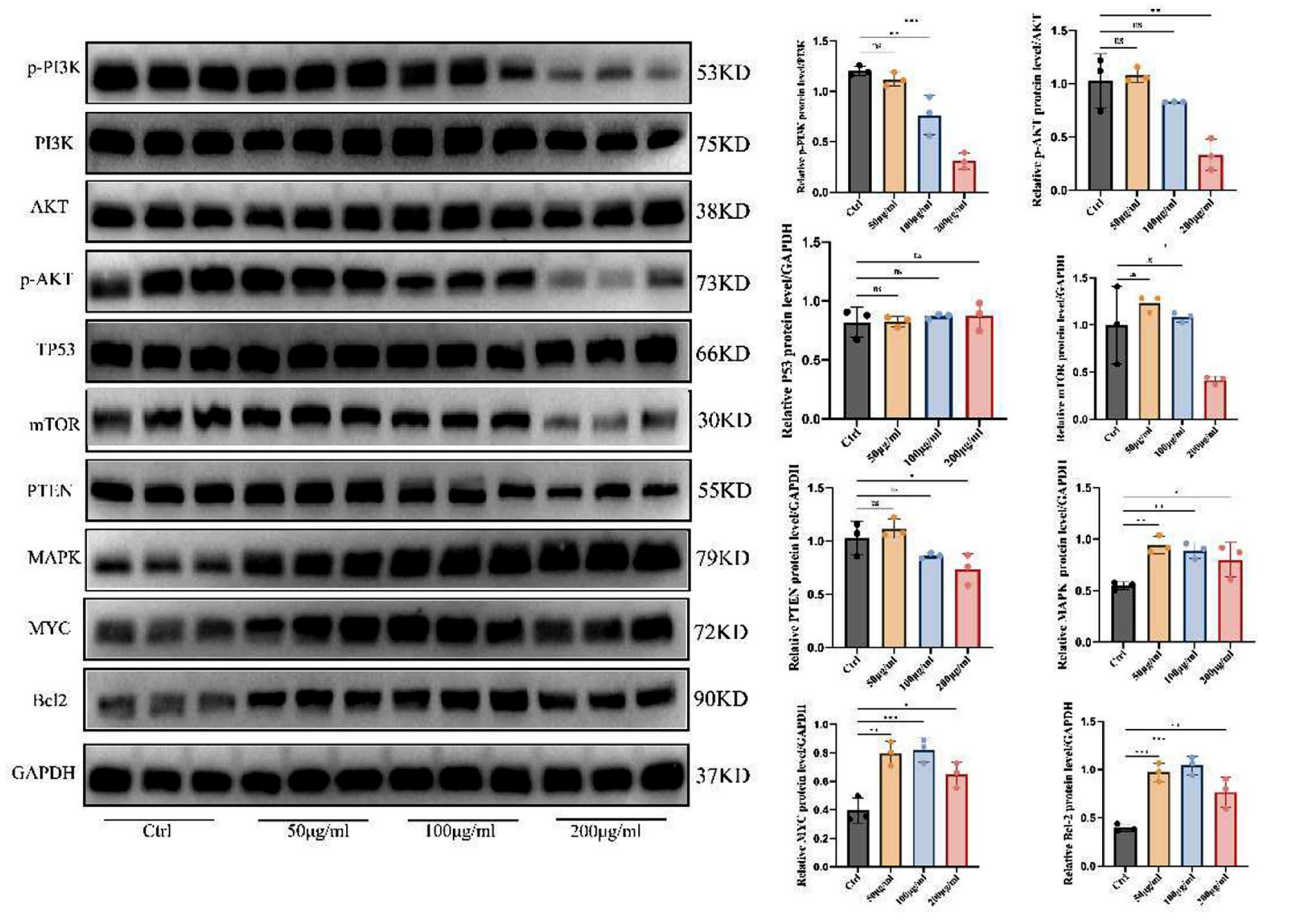
Changes in the content of corresponding proteins in PC3 cells under LWDHD intervention and the statistical graph

**Fig. 10:**
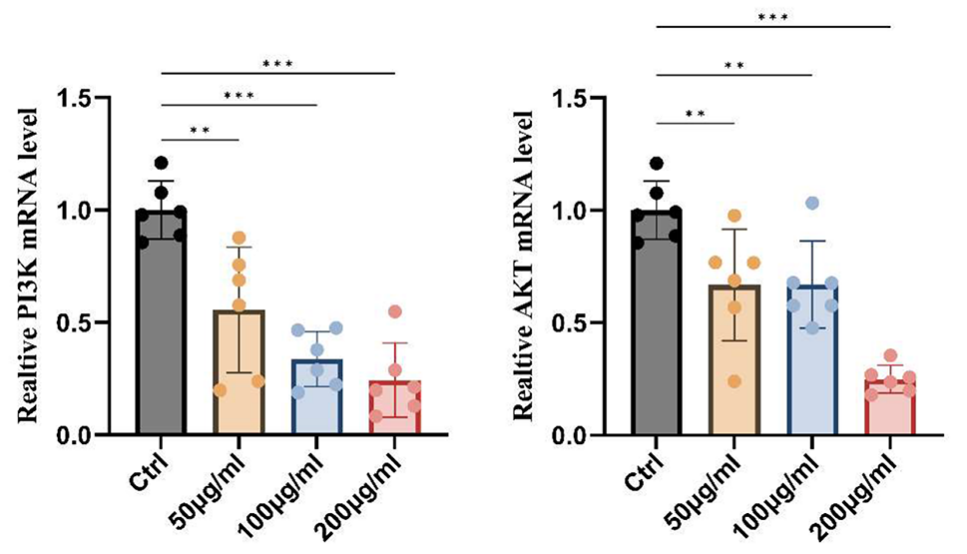
Changes in mRNA levels of PI3K and AKT in PC3 cells under LWDHD intervention

Further, to confirm the mechanism by which LWDHD inhibits prostate cancer value-added, combined with the results of network pharmacology and molecular docking, we performed WB and PCR validation. We found that LWDHD significantly reduced the phosphorylation levels of PI3K and AKT, while there was no significant effect on TP53, mTOR, PTEN, MAPK, MYC, and Bcl2 protein levels. Therefore, we inferred that LWDHD may inhibit prostate cancer proliferation through the PI3K/AKT signaling pathway. We also found that LWDHD could significantly reduce the gene expression levels of PI3K and AKT in PC3 cells, which further verified the correctness of our conjecture. As shown in Figs. 9 and 10.

## Discussion

PCa is a serious life-threatening disease for men [20], and is also a common male malignant tumor in clinical practice. It ranks first among male cancer [1]. Therefore, improving the curative effect of PCa and reducing the disease’s mortality rate is urgent. LWDHD, as a classic Chinese medicine prescription, is often used in cancer treatment.

In this study, we confirmed through cell experiments that LWDHD exhibits inhibitory effects on prostate cancer cell proliferation and effectively suppresses the invasive and migratory capabilities of prostate cancer cells. These findings provide an experimental foundation for exploring the use of LWDHD in the treatment of prostate cancer. Meanwhile, we further use network pharmacology, bioinformatics analysis, and molecular docking to evaluate the anti-tumor effector of LWDHD in PCa and explore the potential mechanism. 74 active ingredients in LWDHD were screened using TCMSP, GeneCards, OMIM and Disgenet databases through network pharmacology. A “drug-ingredient-target” network was established, and 37 ingredients and 99 potential targets for the treatment of PCa were identified. Quercetin, kaempferol and beta-sitosterol correspond to the most targets, which may be the main active ingredients of LWDHD.

Studies have shown that quercetin can prevent the occurrence of PCa by blocking the activity of androgen receptor (AR) and prostate specific antigen (PSA), which can also enhance the influence of other treatment programs on PCa [21, 22]. Kaempferol can promote cell apoptosis, inhibit cell proliferation through an androgen-dependent pathway, and inhibit angiogenic simulation and invasion in PCa [23, 24]. Beta-sitosterol can induce apoptosis of LNCaP human PCa cells by activating sphingomyelin circulation, and beta-sitosterol containing herbs can lead to a decrease in living cells in PCa spheres and prevent tumor growth in mouse xenograft PCa [25, 26]. These may be the main components of LWDHD in the treatment of Pca.

Through the enrichment of GO and KEGG pathways, it is predicted that the potential pathways and targets of LWDHD in the intervention of PCa include phosphatidylinositol 3-kinase/protein kinase B (PI3K/AKT), mitogen activated protein kinase (MAPK), TP53 pathway, MYC, TNF signaling pathway, etc. The PI3K-AKT-mTOR pathway interacts with AR, MAPK, and WNT signaling cascades to promote the growth and drug resistance of PA [27]. MAPKs, a family of serine-threonine kinases, activate AR signaling by enhancing the expression of GAT2 mRNA and inhibiting the ubiquitination and degradation of GATA2 protein, while the synergistic activation of GAT2/AR and AKT pathways is essential for promoting the growth of PCa cells [28–30]. Activation of TP53 promotes a more aggressive phenotype in PCa and increases resistance to treatment [31–33]. MYC transcription factor is one of the most typical driving factors for the occurrence of PCa, and the down-regulation of MEIS1 by MYC mediates the development of PA by increasing the expression of HOXB13 and the activity of AR [34]. Inflammation is believed to increase the risk of PCa, and Tumor necrosis factor (TNF) plays an important role in the inflammatory process, which promotes the development of PCa by promoting the release of cancer-derived soluble factors [35]. Abnormal activation of these targets can promote the proliferation, metastasis, inflammation, angiogenesis and other biological processes of PCa, further aggravating the malignant process of Pca [36]. In conclusion, LWDHD may regulate PI3K/AKT, MAPK, TP53, MYC, TNF and other signaling pathways to participate in the biological processes of cancer proliferation, metastasis, inflammation, angiogenesis and so on, thus inhibiting the development of PCa.

Based on the aforementioned results, incorporating PPI network topology and biological information analyses, it was discovered that MYC was highly expressed in PCa tissues, while CCND1 and MAPK1 exhibited low expression levels. There was a significant correlation between TP53, AKT1, MYC, TNF, CCND1, and MAPK1. This suggested that LWDHD may play a therapeutic role in PCa primarily by regulating genes such as TP53, AKT1, MYC, TNF, CCND1, and MAPK1. Molecular docking results indicated that the main active ingredients of LWDHD, including quercetin, kaempferol, and beta-sitosterol, demonstrated strong binding affinity with core proteins MAPK1, AKT1, P53, MYC, TNF, and CCND1. This evidence substantiates that LWDHD can stably bind to PCa receptor proteins, playing a crucial role. These findings underscore the holistic and integrative nature of traditional Chinese medicine in disease treatment.

Furthermore, cell experiments confirmed that LWDHD inhibits prostate cancer cell proliferation, invasion, and migration. Network pharmacology and molecular docking results, verified by WB, revealed that LWDHD significantly reduces the phosphorylation levels of PI3K and AKT, with no notable impact on TP53, mTOR, MAPK, MYC, and other protein levels. Additionally, PCR experiments showed that LWDHD significantly decreases PI3K and AKT gene expression in PC3 cells. Therefore, it is inferred that LWDHD may inhibit prostate cancer proliferation through the PI3K/AKT signaling pathway.

The PI3K/AKT signaling pathway regulates cellular metabolism, tumor development, growth, proliferation, metastasis, and cytoskeletal reorganization [37]. In prostate cancer, activation of this pathway reduces the cells’ dependence on growth factors and nutrients, enabling indefinite tumor cell proliferation [27, 38]. This unlimited proliferation property is a key feature of tumor development [39]. Numerous studies have demonstrated the critical role of the PI3K/AKT signaling pathway in prostate cancer, including inducing apoptosis [40], causing cell cycle arrest [41], and regulating EMT progression [42] to inhibit tumor cell growth, migration, and invasion [43].

Therefore, therapeutic strategies targeting the PI3K/AKT signaling pathway are important in tumor therapy [44, 45]. By inhibiting the activity of PI3K or AKT, the proliferation signals of tumor cells can be blocked, thus achieving the purpose of tumor treatment [46]. A number of inhibitors targeting the PI3K/AKT signaling pathway have been developed and applied to tumor therapy with certain efficacy [47]. At the same time, traditional Chinese medicine and natural products are a huge treasure house with good anti-tumor effect, which is worth further exploration and research [48, 49]. This study lays a foundation for further validating the mechanism of action of LWDHD in treating prostate cancer and provides new insights for research on traditional Chinese medicine in the treatment of prostate cancer.

### Electronic supplementary material

Below is the link to the electronic supplementary material.

Supplementary Material 1

## Data Availability

The original contributions presented in the study are included in the article; further inquiries can be directed to the corresponding authors upon reasonable request.
