# Supplementary material for: Network pharmacology and experimental validation to explore the role and potential mechanism of Liuwei Dihuang Decoction in prostate cancer
Source: BMC Complement Med Ther. 2024 Jul 26;24:284. doi: 10.1186/s12906-024-04572-5 (PMC11282786; doi:10.1186/s12906-024-04572-5)

Supplementary material for Western Blotting

**Fig. 10**

GAPDH


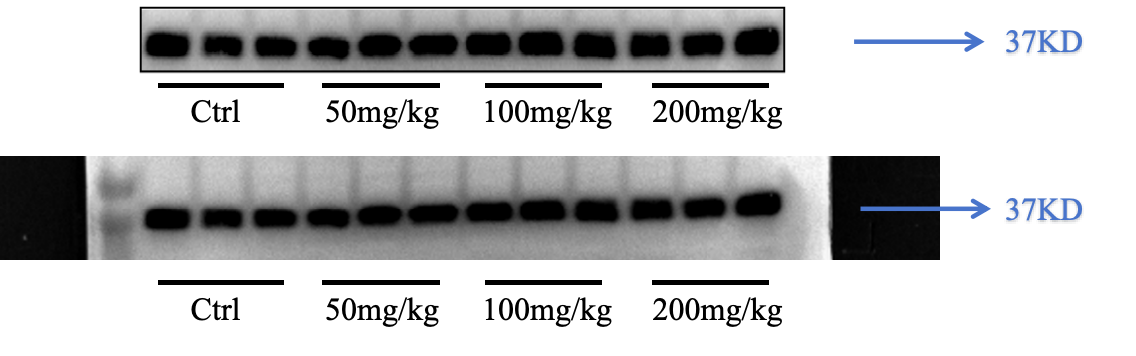


1. PI3k


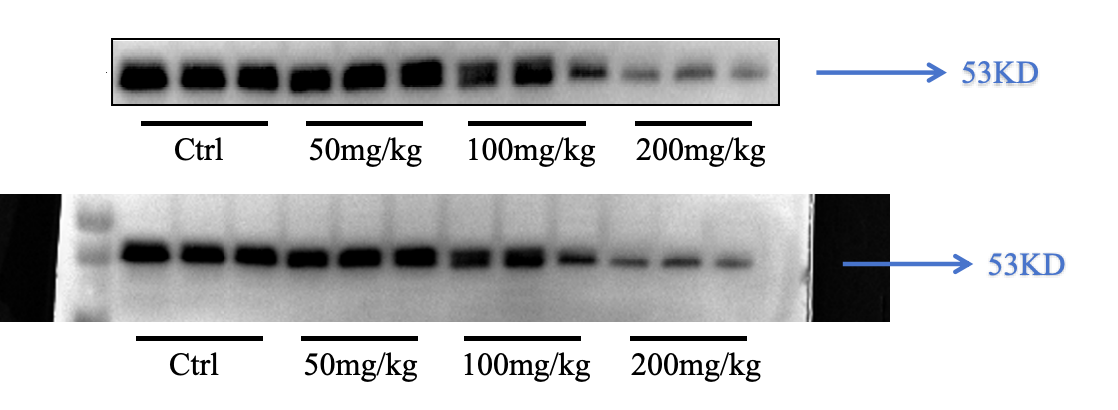


PI3K


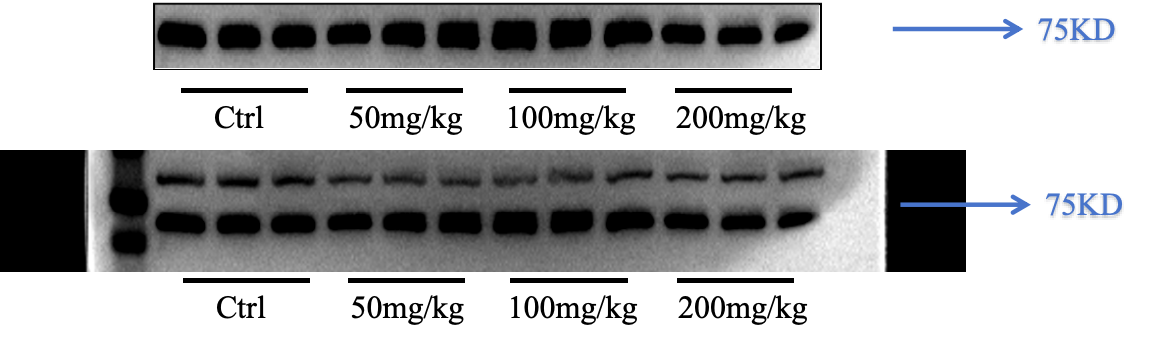


AKT


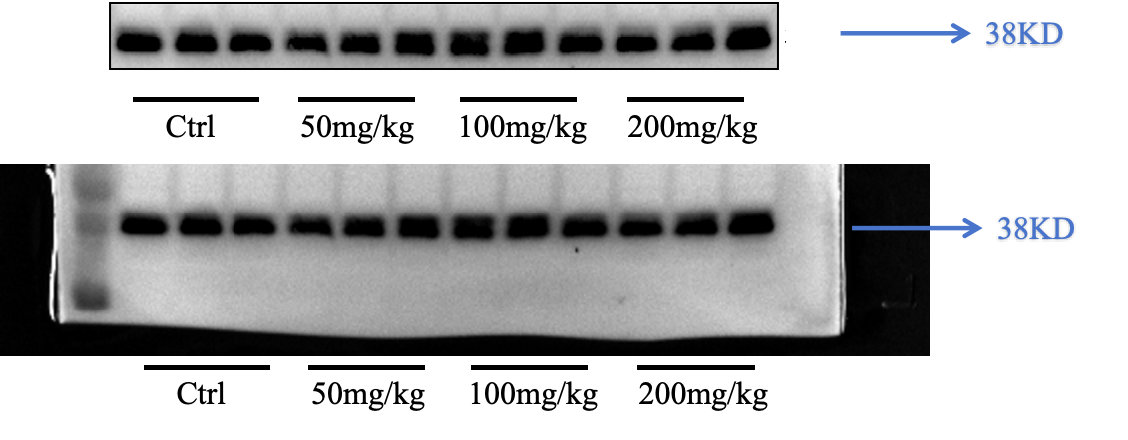


P-AKT


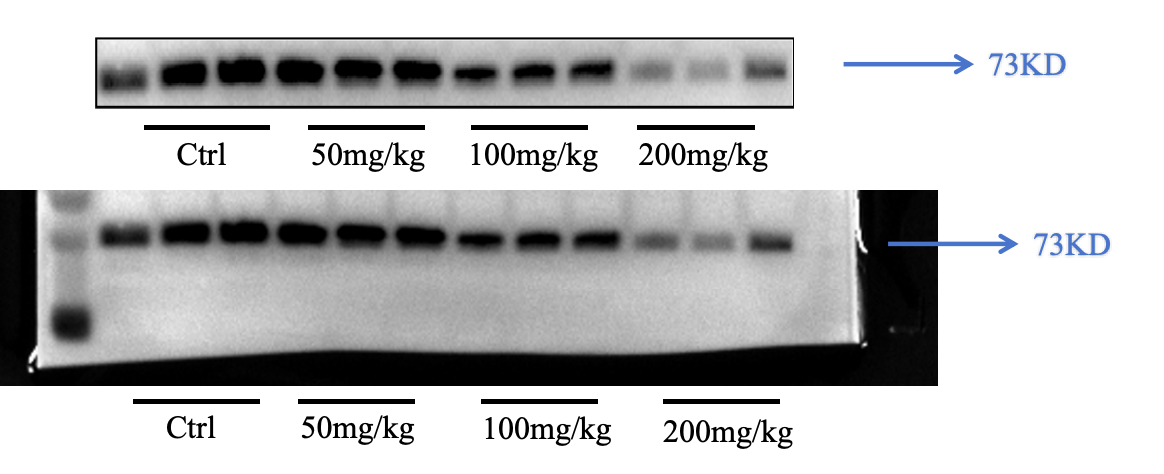


TP53


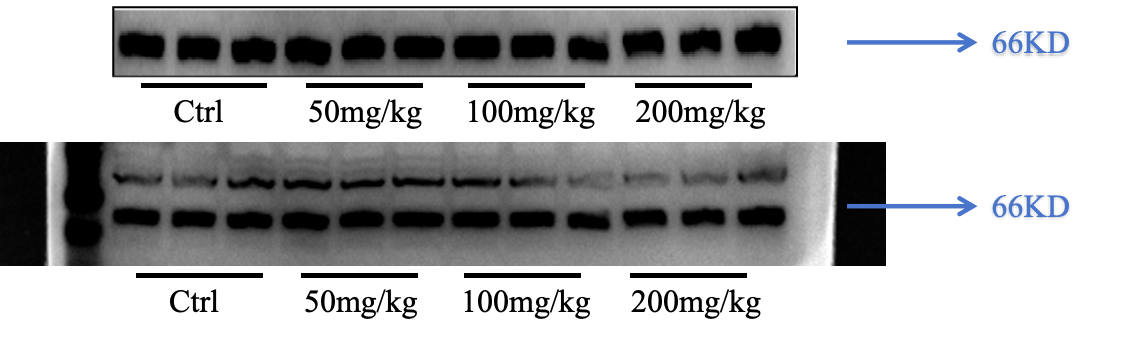


mTOR


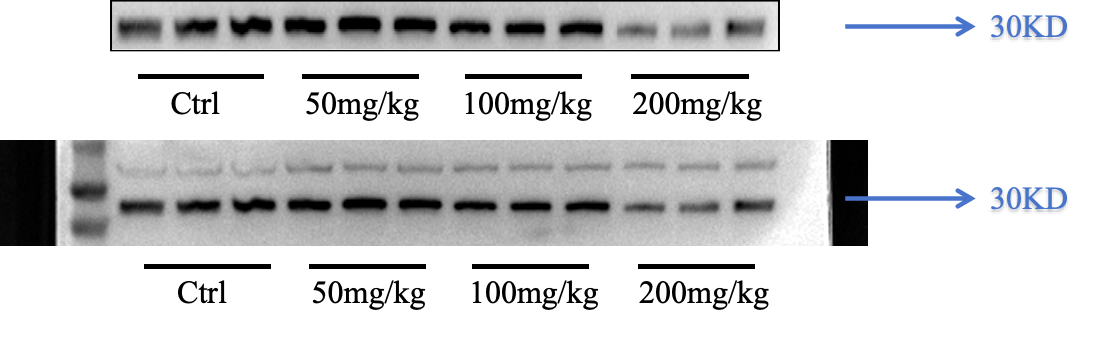


PTEN


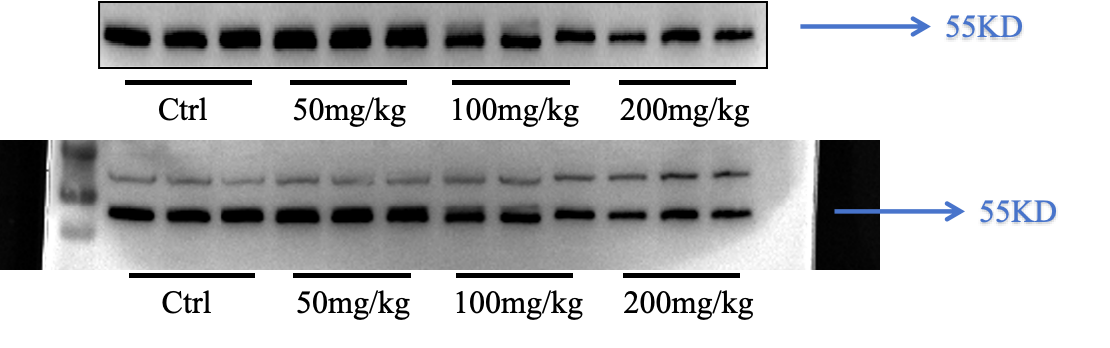


MAPK


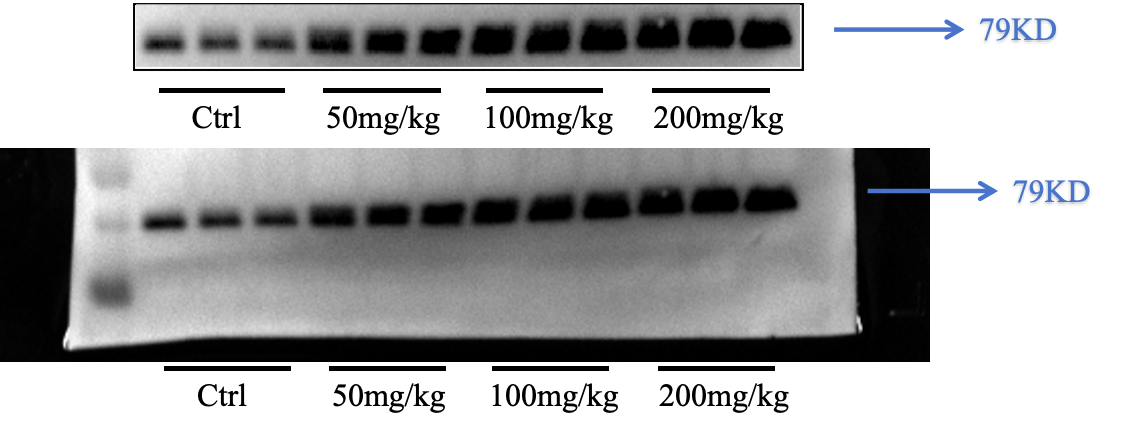


MYC


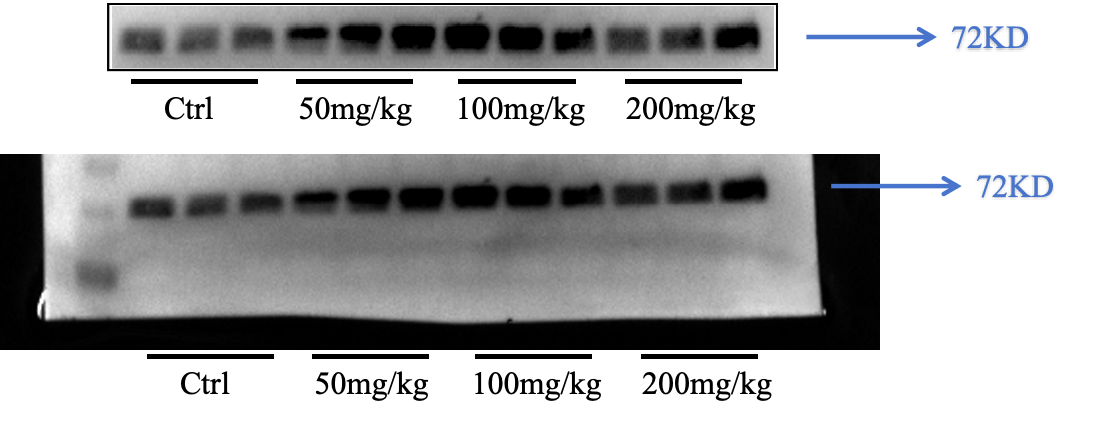


Bcl-2


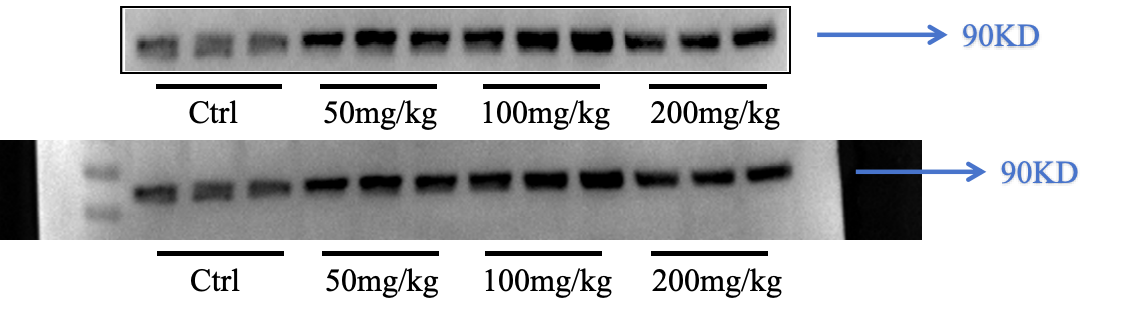

Supplement: Supplementary file 1 — Supplementary Material 1 [file 12906_2024_4572_MOESM1_ESM.docx]
